# The relationship between spinal alignment and activity of paravertebral muscle during gait in patients with adult spinal deformity: a retrospective study

**DOI:** 10.1186/s12891-022-06121-y

**Published:** 2023-01-03

**Authors:** Tomoyuki Asada, Kousei Miura, Hideki Kadone, Kotaro Sakashita, Toru Funayama, Hiroshi Takahashi, Hiroshi Noguchi, Kosuke Sato, Fumihiko Eto, Hisanori Gamada, Kento Inomata, Masao Koda, Masashi Yamazaki

**Affiliations:** 1 Department of Orthopaedic Surgery, Faculty of Medicine, University of Tsukuba, 1-1-1 Tennodai, Tsukuba, Ibaraki 305-8575 Japan; 2 Center for Innovative Medicine and Engineering, University of Tsukuba Hospital, 1-1-1 Tennodai, Tsukuba, Ibaraki 305-8575 Japan

**Keywords:** Gait analysis, Adult spinal deformity, Paravertebral muscle, Integrated EMG, Dynamic spinal parameters

## Abstract

**Background:**

Spinal alignment in patients with adult spinal deformity (ASD) changes between rest and during gait. However, it remains unclear at which point the compensated walking posture breaks down and how muscles respond. This study used time-synchronized electromyography (EMG) to investigate the relationship between dynamic spinal alignment and muscle activity during maximum walking duration to reveal compensation mechanisms.

**Methods:**

This study collected preoperative three-dimensional gait analysis data from patients who were candidates for corrective surgery for ASD from April 2015 to May 2019. We preoperatively obtained dynamic spinal alignment parameters from initiation to cessation of gait using a motion capture system with time-synchronized surface integrated EMG (iEMG). We compared chronological changes in dynamic spinal alignment parameters and iEMG values 1) immediately after gait initiation (first trial), 2) half of the distance walked (half trial), and 3) immediately before cessation (last trial).

**Results:**

This study included 26 patients (22 women, four men) with ASD. Spinal sagittal vertical axis distance during gait (SpSVA) increased over time (first vs. half vs. last, 172.4 ± 74.8 mm vs. 179.9 ± 76.8 mm vs. 201.6 ± 83.1 mm; *P* < 0.001). Cervical paravertebral muscle (PVM) and gluteus maximus activity significantly increased (*P* < 0.01), but thoracic and lumbar PVM activity did not change. Dynamic spinal alignment showed significant correlation with all muscle activity (cervical PVM, r = 0.41–0.54; thoracic PVM, r = 0.49–0.66; gluteus maximus, r = 0.54–0.69; quadriceps, r = 0.46–0.55) except lumbar PVM activity.

**Conclusion:**

Spinal balance exacerbation occurred continuously in patients with ASD over maximum walking distance and not at specific points. To maintain horizontal gaze, cervical PVM and gluteus maximus were activated to compensate for a dynamic spinal alignment change. All muscle activities, except lumbar PVM, increased to compensate for the spinal malalignment over time.

## Introduction

Adult spinal deformity (ASD) is a primary disorder causing lower back pain during gait, characterized by spinal malalignment in radiographic assessment. The prevalence of ASD is increasing with societal aging [1–3], and the disability and social financial burden of ASD are well-studied [4, 5]. However, the pathophysiology of ASD remains unclear because spinal balance is a dynamic concept that is difficult to assess in clinical practice [5–7].

Recently, the analysis of dynamic spinal alignment and compensatory mechanisms have been reported for maintaining standing and walking positions in patients with ASD [8–13]. Walking load worsens spinal balance compared to a static standing posture [14], and spinal alignment in patients with ASD becomes unbalanced during gait [15]. Walking disrupts the compensatory mechanisms during standing posture and causes discrepancies in spinal alignment. Recent studies have reported the importance of paravertebral muscle assessment during this spinal alignment change [8, 10, 12, 16]. As sagittal spinal unbalance occurs during walking, the trunk muscle group tries to respondto maintain spinal balance [5, 7]. However, most gait analyses investigated patients’ short walking with or without eliciting symptoms and measured muscle activity using the cross-sectional area of the paravertebral muscles from imaging studies as a surrogate [8, 10, 12]. To the best of our knowledge, few studies have investigated at which point during maximum walking distance the posture collapse, namely soon after walking starts, soon before the patient stops, or when it gradually worsens. Furthermore, the muscle’s actual activity response as a compensation mechanism remains unknown.

Our study aimed to investigate the walking posture and muscle activity of patients with ASD during their maximum walking duration limited by symptoms for evaluating the chronological changes in spinal alignment and muscle activity during gait. We hypothesized that all segments of the paraspinal muscles would be activated in accordance with a forward-leaning posture during gait and activity would decrease near the end of gait.

## Methods

### Study design and inclusion criteria

This study collected data from patients with ASD who were candidates for corrective surgery and gave written informed consent for three-dimensional (3D) gait analysis from April 2015 to May 2019. The inclusion criteria were as follows: 1) patients with ASD over 50 years, 2) who suffered from lower back pain during gait, and 3) were able to walk over 10 m without support. Radiographic criteria were spinal parameters related to sagittal malalignment according to the SRS-Schwab ASD classification [17] as follows: pelvic incidence minus LL (PI-LL) > 10°; sagittal vertical axis (SVA) > 4 cm; and/or pelvic tilt (PT) > 20°. Exclusion criteria were as follows: 1) unable to walk over 25 m without rest and 2) able to walk more than 15 min. This study was conducted with approval from our local ethics committee (Tsukuba Clinical Research and Development Organization) (approval number: H26-144) in accordance with the principles of the Declaration of Helsinki.

### Data collection

We collected background data of the patients, including sex, height, and body weight. Radiographical assessments included whole spine parameters collected digitally in an outpatient clinic. All patients were asked to stand in their usual posture and look straight ahead in the radiographic exam.

The following spinal parameters were measured from the entire spinal X-ray imaging: sagittal vertical axis distance (SVA), thoracic kyphosis (TK), LL, PT, pelvic incidence (PI), T1 pelvic angle (TPA), coronal Cobb angle of the thoracolumbar and lumbar scoliosis (Cobb; negative indicating convex to the left), and coronal balance (C7-CSVL; the distance between a C7 plumb line and the center sacral vertical line; negative indicating the patient’s left).

### 3D gait analysis

**Fig. 1:**
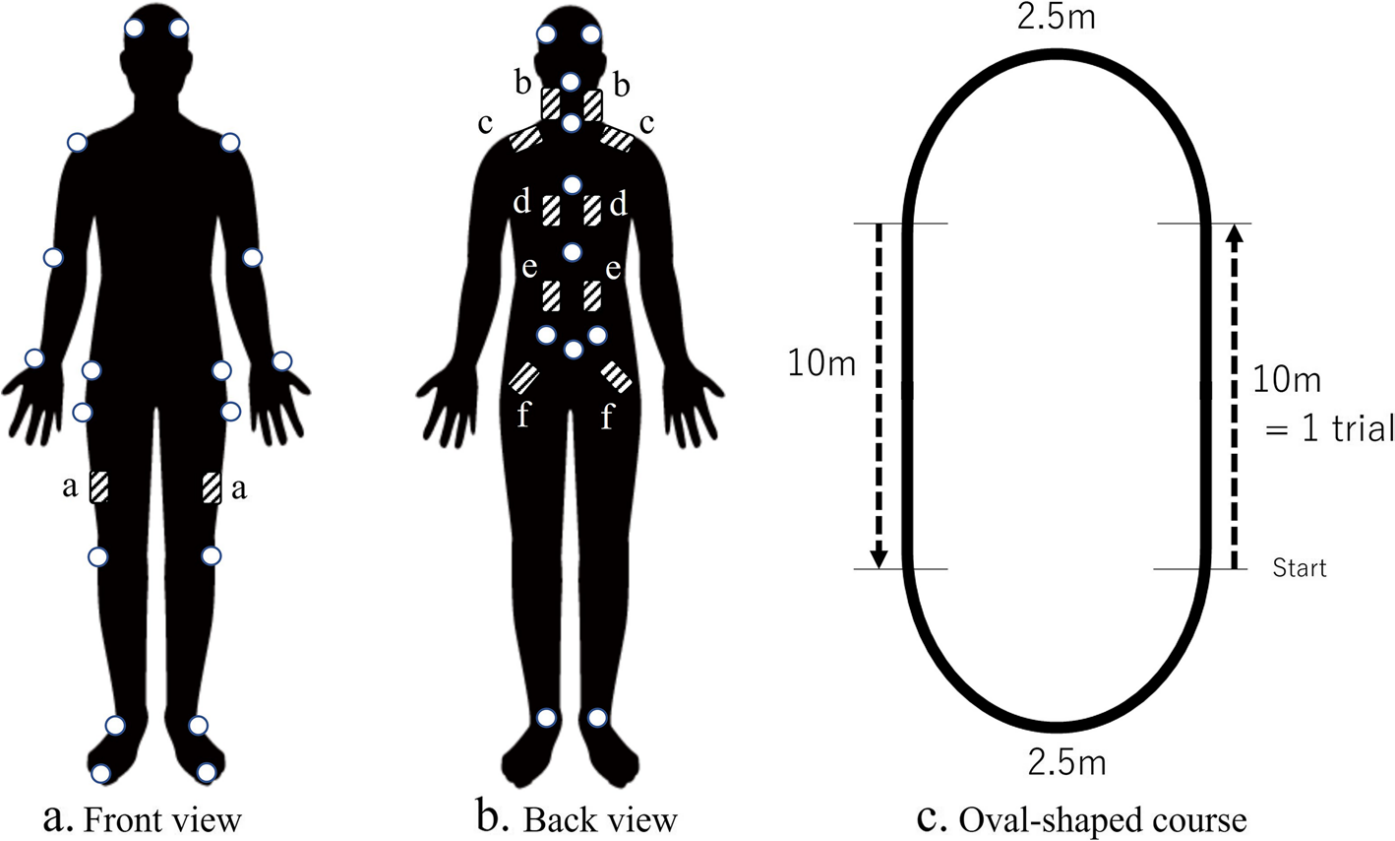
Reflective markers (white circle), electrodes (shaded squares) placement, and oval-shaped course. **a**) Markers and electrodes in the front. Electrodes: a, Quad. **b**) Markers and electrodes in the back. Electrodes: b, cervical PVM; **c**, trapezius m.; **d**, thoracic PVM; **e**, lumbar PVM; **f**, Gmax. c) Oval-shaped course. Gait analysis was conducted only for the straight part of the course. *Quad* quadriceps muscle, *PVM* paravertebral muscle, *Gmax* gluteal maximum muscle

**Fig. 2:**
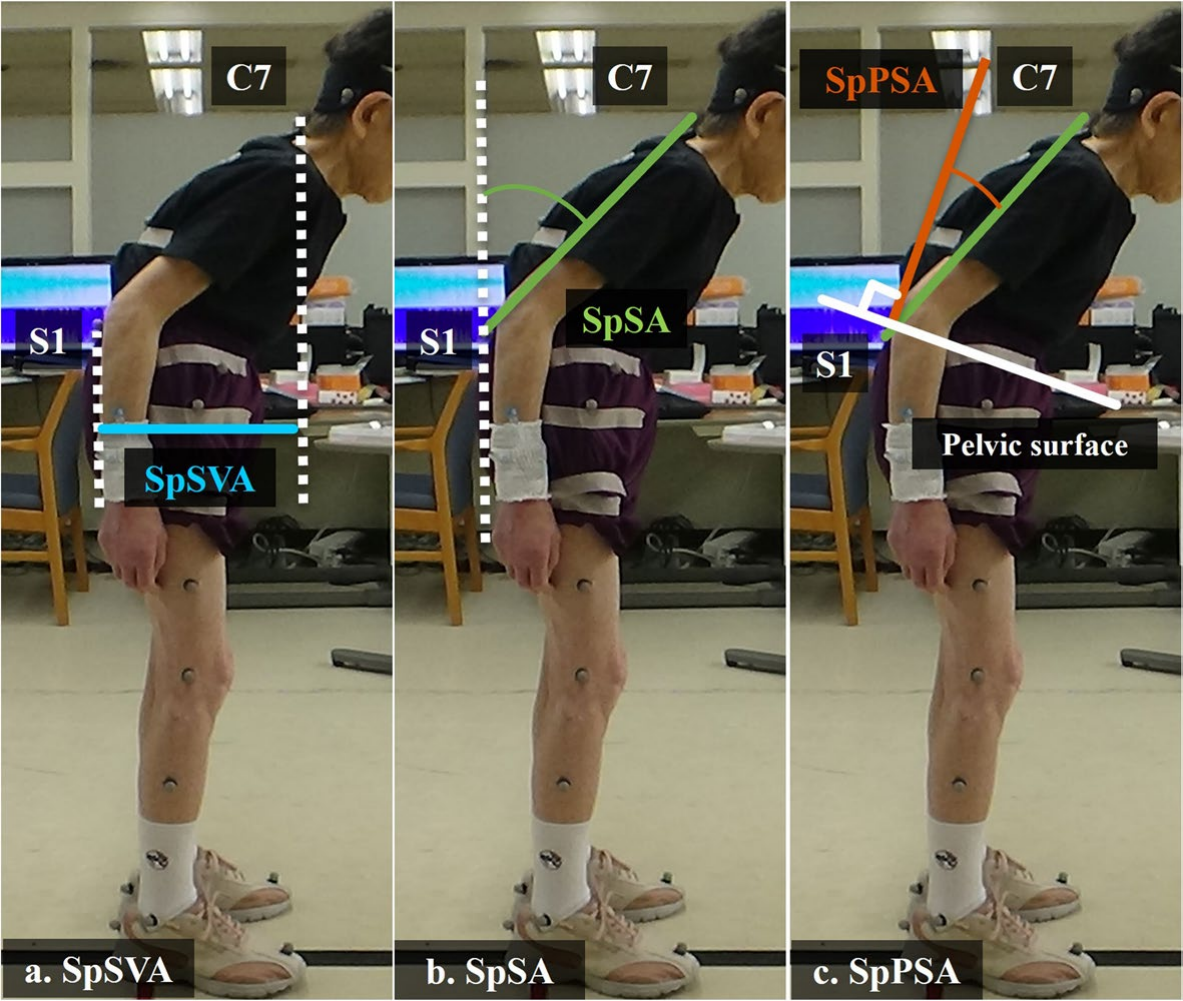
**a**. SpSVA was defined as the distance between the plumb lines (dotted lines) from C7 and S1 markers. **b**. SpSA was defined as the angle between the plumb line from the S1 marker (the dotted line) and the line connecting C7 and S1 markers (the green line). c. SpPSA was measured as the angle between the plumb line (the orange line) to the pelvic surface (the white solid line) and the line connecting C7 and S1 markers (the green line). The pelvic surface was created by bilaterally connecting the ASIS and the PSIS. All angles were projected from three-dimensional angles to a 2D sagittal surface. *SpSVA* spinal sagittal vertical axis distance, *SpSA* spinal sagittal angle, *SpSVA* spinal sagittal vertical axis distance, *ASIS* anterior superior iliac spine, *PSIS* posterior superior iliac spine

3D gait analyses included dynamic spinal parameters and integrated EMG (iEMG) analysis. We conducted motion capture using a Nexus motion capture system (Vicon, Oxford, UK) comprising 16 cameras and reflective makers attached to the spinal spinous processes, pelvis, and lower limbs of the patients (Fig. 1 a and b). The markers were applied over the clothes in the trunk area for patient privacy. The clothing was tightened to the extent possible without disrupting the walking posture to minimize the potential error by soft tissue and clothes. Based on the Vicon plug-in gait marker set (Vicon Nexus software version 2.2.3), reflective markers were pasted on the lower limb to evaluate the gait cycle. We instructed patients to walk at a comfortable speed repeatedly around a 25-m oval-shaped course in the examination room for their maximum duration (Fig. 1 c). We defined the 10-m straight part in the oval shape as a trial. When lower back pain was prohibitive and the patient judged the walking distance was their maximum, they could stop walking upon request and the gait analysis recording ended. If their maximum walking duration was over 15 min, we stopped the exam and excluded the patients as those who could walk more than 15 min had less disability due to attributed to back pain. They were unlikely to have surgery prescribed for them in our institution. Spinal SVA (SpSVA) was measured as the horizontal distance between C7 to S1 markers (Fig. 2a). Spinal sagittal angle (SpSA) was defined as sagittal projected angles between the spinal alignment line created by markers and the plumb line (Fig. 2b). The spinal alignment line was from the markers on C7 to those on S1, and the pelvic surface from the markers on the anterior superior iliac spine (ASIS) to those on the posterior superior iliac spine (PSIS) (Fig. 2c). To measure spinal balance not affected by the pelvis and lower extremity compensation, spinal-pelvic sagittal angle (SpPSA) was defined as the sagittal projected angle from the spinal alignment line and a plumb line to the pelvic surface (Fig. 2C). These data were initially averaged per a walk cycle. The average values for each walking cycle were subsequently collected only for walking along a straight line, and these values were calculated as the average values of the parameters in one trial adjusted by the number of steps.

Time-synchronized EMG analysis was conducted by the TringoTM Lab wireless surface EMG system (Delsys Inc, Boston, MA, USA). We placed wireless surface electrodes on the skin surfaces of each muscle segment: the cervical paravertebral muscles (PVM) between C2-C7 spinous process, the trapezius on the trapezius muscle, the thoracic PVM between the T6-T12 spinous process, the lumbar PVM between the L3-L5 spinous process, the gluteus maximus muscle (Gmax) on the buttock, and the quadriceps (Quad) on the vastus lateralis (Fig. 1 a and b). The placements were performed upon palpation by one or two spine surgeons. Muscle activity was assessed using an iEMG. EMG data were first filtered with a 30–400 Hz bandpass and rectified and locally integrated using a 200-ms moving window to obtain an iEMG profile. This iEMG profile was then divided into steps according to the height of the toe and heel markers; 10 m walking was considered as one trial, and the average of iEMG during one trial was calculated (Fig. 1c). This processing was performed by MATLAB 8.4 (MathWorks, Natick, MA, USA). We obtained mean values of dynamic spinal parameters and iEMG over three trials during gait analysis: 1) a trial after gait initiation (first trial), 2) a trial before gait cessation (last trial) and 3) a trial at the midpoint of the gait duration (half trial).

### Statistical analysis

All quantitative values are described as mean ± standard deviation (SD). iEMG values were log-transformed for normalization prior to statistical analysis. Shapiro–Wilk analysis was used for validation of normal distribution. Chronological changes in dynamic spinal parameters and iEMG were examined with a one-way repeated-measure analysis of variance (ANOVA), followed by the Bonferroni test as post hoc analysis. Correlation analysis between the dynamic spinal parameters and iEMG was performed using Pearson’s correlation coefficient (r). All the statistical analyses were performed with R (version 4.0.2, https://www.R-project.org/.) A P-value < 0.05 was considered statistically significant.

## Results

Twenty-six patients (22 women and four men) were included in this study. Patient demographic data are shown in Table 1.

**Table 1:** Demographic data (*n* = 26)

		Mean	SD
Age	(y.o.)	68.7	7.6
BMI	(kg/m^2^)	22.4	2.9
C7CSVL	(cm)	4.2	26.3
Cobb	(°)	24.8	18.4
C7SVA	(mm)	107.3	65.1
TK	(°)	22.3	16.4
LL	(°)	9.1	21.0
PT	(°)	33.2	14.2
PI	(°)	46.8	12.0
TPA	(°)	36.4	15.7
PI-LL	(°)	37.6	23.0

*BMI* Body mass index, *C7CSVL* C7 plumb line to central sacral vertical line distance, *Cobb* main Cobb angle, *C7SVA* C7 to sacral sagittal vertical axis, *TK* thoracic kyphosis, *LL* lumbar lordosis, *PT* pelvic tilt, *PI* pelvic incidence, *TPA* T1 pelvic angle, *PI-LL* PI minus LL, *SD* standard deviation

**Fig. 3:**
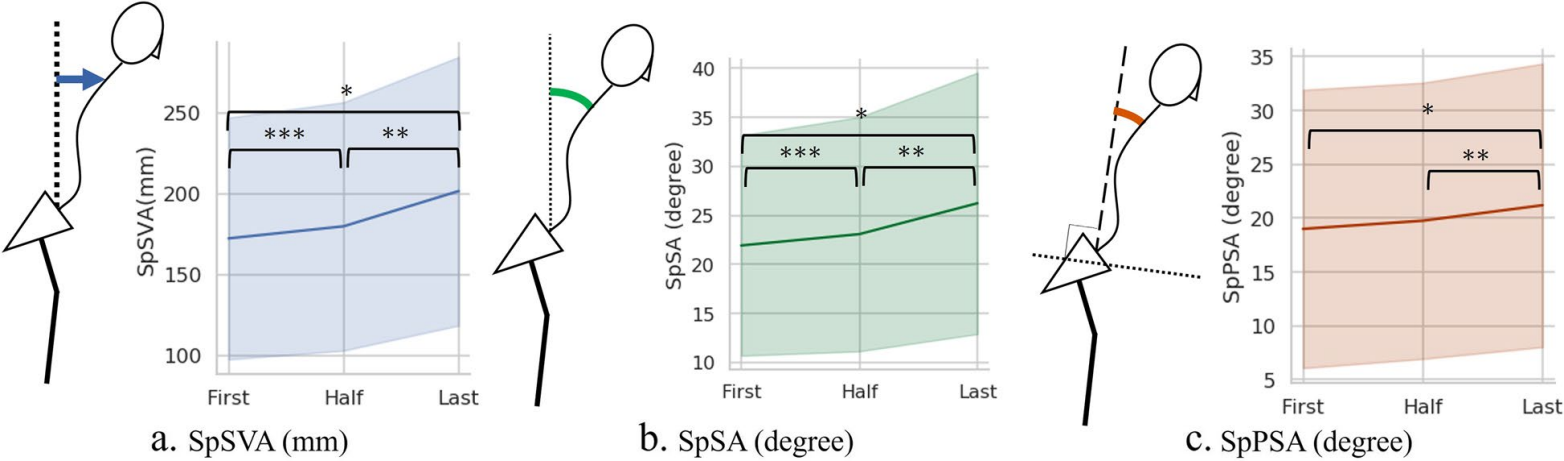
**a**. SpSVA increased significantly over time during walking. **b**. SpSA increased significantly over time during walking. **c**. SpPSA increased significantly between half and last trials. *SpSVA* spinal sagittal vertical axis distance, *SpSA* spinal sagittal angle, *SpSVA* spinal sagittal vertical axis distance. *; *P* < 0.001 in repeated-measure ANOVA analysis between first, half and last trial. **; *P* < 0.001 in post hoc analysis between each group. ***; *P* < 0.05 in post hoc analysis between each group

**Fig. 4:**
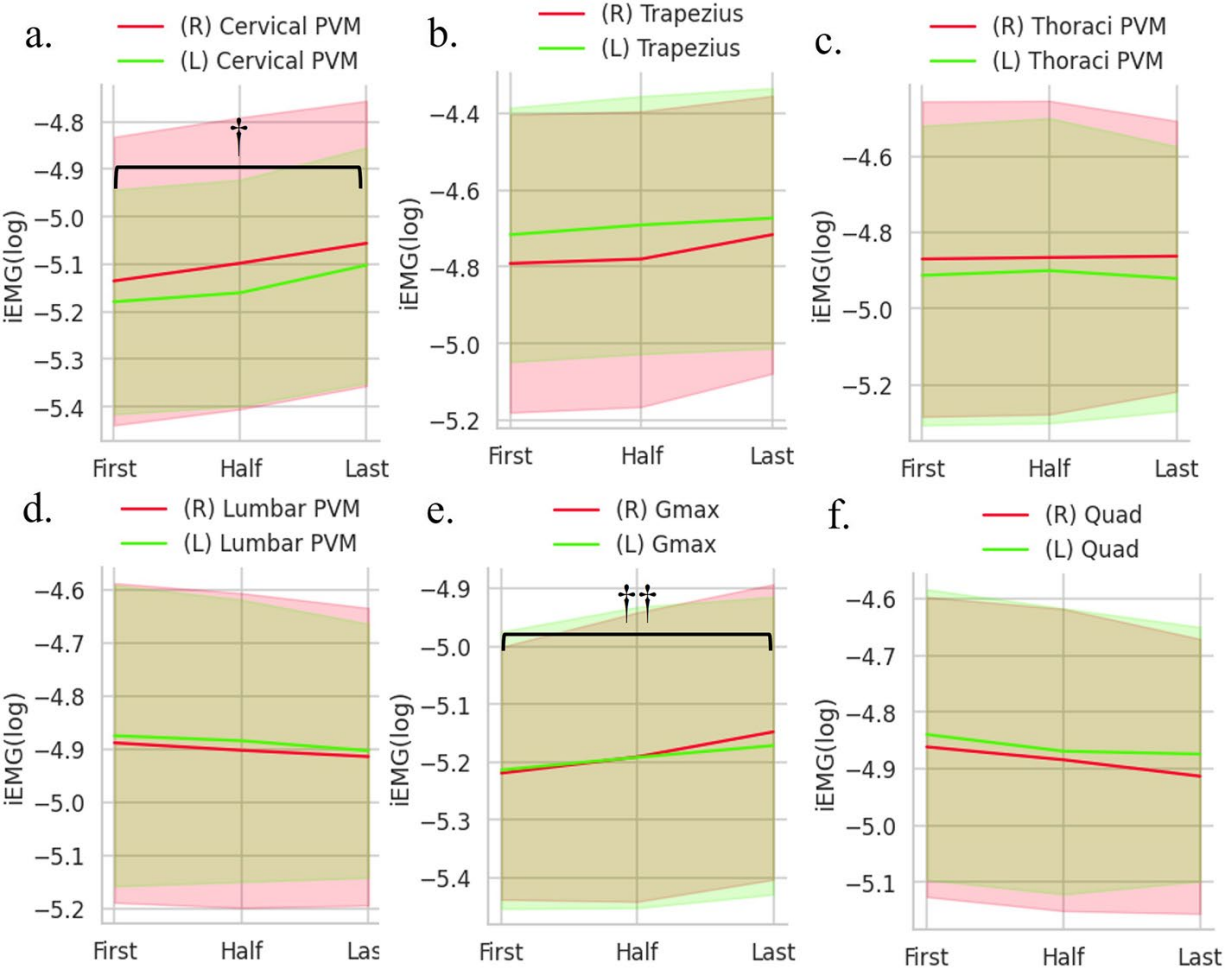
These figures indicate each muscle’s iEMG change over time during walking. The red line indicates the right side (R), and the green line indicates the left side (L). **a**. Cervical PVM. **b**. Trapezius muscle. **c**. Thoracic PVM. **d**. Lumbar PVM. **e**. Gmax. **f**. Quad. *iEMG* integrated electromyography, *PVM* paravertebral muscle, *Gmax* gluteus maximus, *Quad* quadriceps. †; *P* < 0.05 in repeated-measure ANOVA analysis between first, half, and last trials. ††; *P* < 0.05 in repeated-measure ANOVA analysis for iEMG of the right Gmax

SpSVA, SpSA, and SpPSA significantly increased during gait (*P* < 0.001, Table 2). SpSVA increased over time (first vs half, 172.4 ± 74.8 vs 179.9 ± 76.8, *P* = 0.034; half vs last, 179.9 ± 76.8 vs 201.6 ± 83.1, *P* < 0.001, Fig. 3A). SpSA also increased over time (first vs half, 21.9 ± 11.2 vs 23.1 ± 11.9, *P* = 0.028; half vs last, 23.1 ± 11.9 vs 26.2 ± 13.3, *P* < 0.001, Fig. 3B). SpPSA increased significantly, especially from half to last trial (first vs half, 19.0 ± 12.9 vs 19.8 ± 12.8, *P* = 0.097; half vs last, 19.8 ± 12.8 vs 21.2 ± 13.2, *P* = 0.0017, Fig. 3C). The analysis of each muscle (Fig. 4) revealed significant increases in cervical PVM (right, *P* = 0.0029; left, *P* = 0.0010). The changes in other muscles were not significant except in the right gluteus maximus (*P* = 0.015, Fig. 4e).

**Table 2:** Chronological change of dynamic spinal parameters and muscular activity

			**First**		**Half**		**Last**			
			**Mean**	**SD**	**Mean**	**SD**	**Mean**	**SD**	***P*-value**	**Post hoc analysis**
SpSVA	(mm)		172.4	74.8	179.9	76.8	201.7	83.1	< 0.001	FvsH, HvsL
SpSA	(°)		21.9	11.2	23.1	11.9	26.2	13.3	< 0.001	FvsH, HvsL
SpPSA	(°)		19.0	12.9	19.8	12.8	21.2	13.2	< 0.001	HvsL
Cervical PVM	(mV)	Rt	-5.14	0.30	-5.10	0.31	-5.06	0.30	0.003	FvsH, FvsL
		Lt	-5.18	0.24	-5.16	0.24	-5.10	0.25	0.001	FvsH, FvsL
Trapezius	(mV)	Rt	-4.79	0.39	-4.78	0.38	-4.72	0.36	0.205	
		Lt	-4.72	0.33	-4.69	0.34	-4.67	0.34	0.320	
Thoracic PVM	(mV)	Rt	-4.87	0.41	-4.87	0.41	-4.86	0.36	0.877	
		Lt	-4.91	0.39	-4.90	0.40	-4.92	0.35	0.568	
Lumbar PVM	(mV)	Rt	-4.89	0.30	-4.90	0.30	-4.91	0.28	0.451	
		Lt	-4.87	0.28	-4.88	0.27	-4.90	0.24	0.460	
Gmax	(mV)	Rt	-5.22	0.22	-5.19	0.25	-5.15	0.26	0.016	FvsL
		Lt	-5.21	0.24	-5.19	0.26	-5.17	0.26	0.117	
Quadriceps	(mV)	Rt	-4.86	0.26	-4.88	0.27	-4.91	0.24	0.041	
		Lt	-4.84	0.26	-4.87	0.25	-4.87	0.22	0.199	

Muscular activity shown by integrated electromyography (iEMG), which is log transformed for normalized deviation

*SpSVA* Spinal sagittal vertical axis distance*, SpSA* Spinal sagittal angle*, SpPSA* Spinal-pelvic angle, *Cervical PVM* cervical paravertebral muscle, *Thoracic PVM* thoracic paravertebral muscle, *Lumbar PVM* lumbar paravertebral muscle, *Gmax* gluteus maximus, *F* first trial, *H* half trial, *L* last trial

**Fig. 5:**
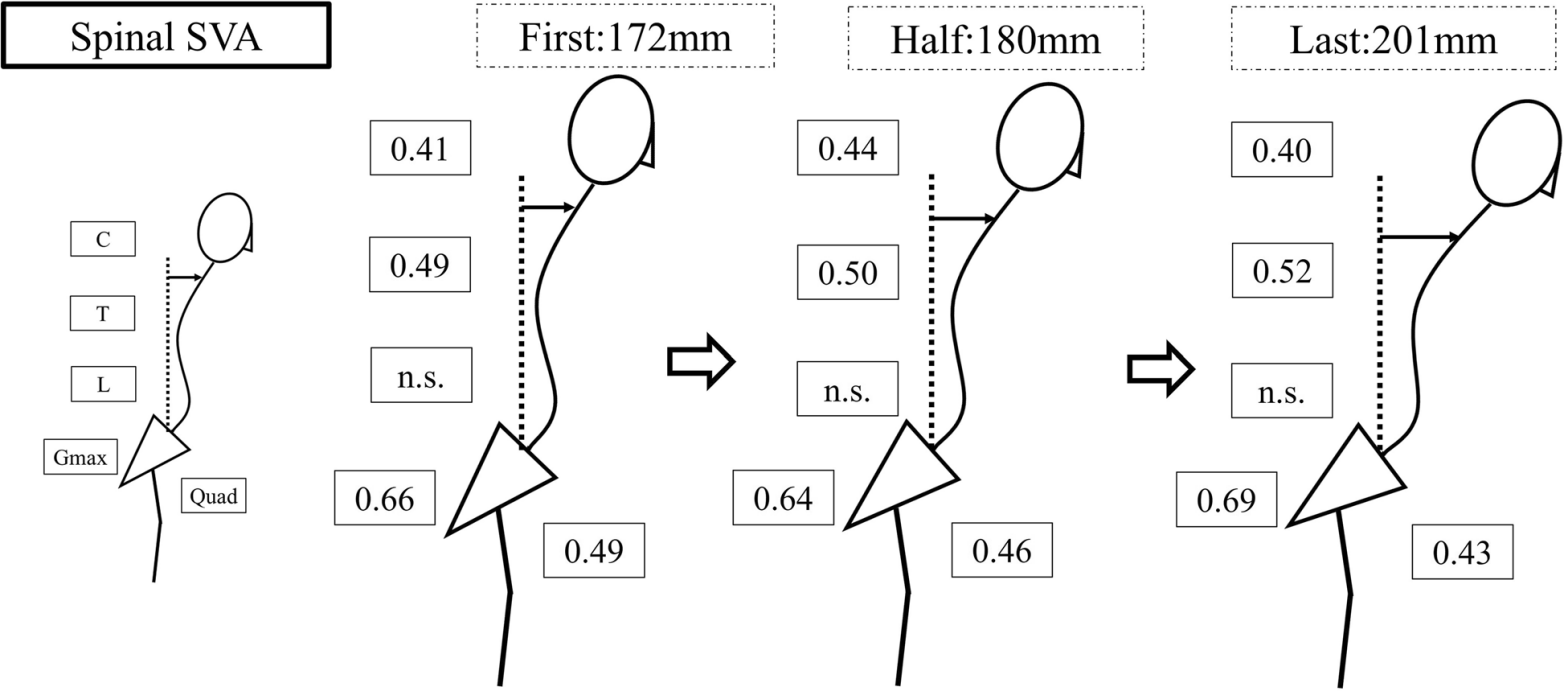
The correlation coefficients between SpSVA and each muscle at first, half, and last trials. Cervical PVM, thoracic PVM, Gmax, and Quad showed moderate correlations with SpSVA. *SpSVA* spinal sagittal vertical axis distance, *PVM* paravertebral muscle, *C* cervical PVM, *T* thoracic PVM, *L* lumbar PVM, *Gmax* gluteus maximus muscle, *Quad* quadriceps muscle, *n.s.* not significant indicating *P* > 0.05 in Pearson correlation coefficients

**Fig. 6:**
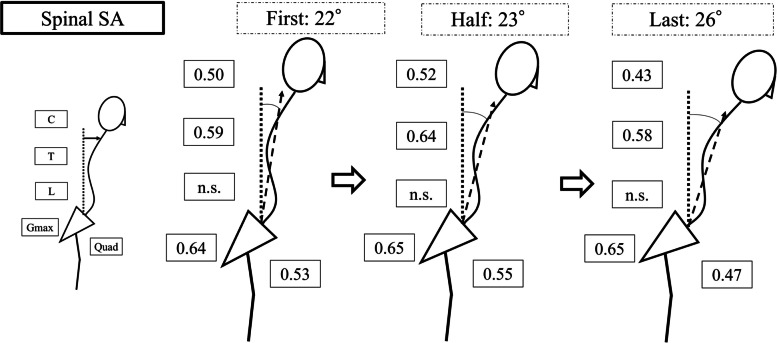
The correlation coefficients between SpSA and each muscle at first, half, and last trials. Cervical PVM, thoracic PVM, Gmax, and Quad showed moderate correlations with SpSA. *SpSA* spinal sagittal angle, *PVM* paravertebral muscle, *C* cervical PVM, *T* thoracic PVM, *L* lumbar PVM, *Gmax* gluteus maximus muscle, *Quad* quadriceps muscle, *n.s.* not significant indicating *P* > 0.05 in Pearson correlation coefficients

**Fig. 7:**
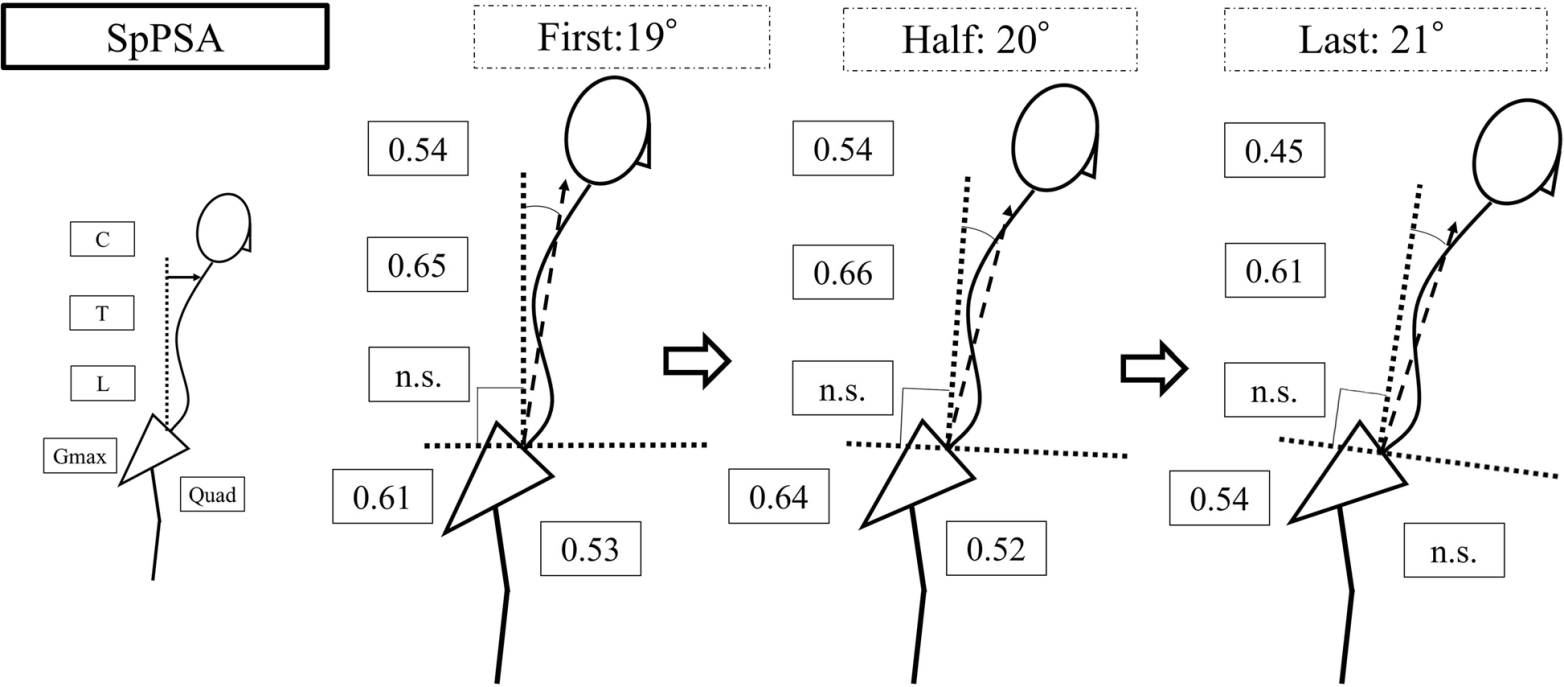
The correlation coefficients between SpPSA and each muscle at first and half trials. Cervical PVM, thoracic PVM, Gmax, and Quad showed moderate correlations with SpPSA, but the correlation between Quad and SpPSA was not significant in the last trial. *SpPSA* spinal-pelvic sagittal angle, *C* Cervical PVM, *T* Thoracic PVM, *L* Lumbar PVM, *Gmax* gluteus maximus muscle, *Quad* quadriceps muscle, *n.s.* not significant indicating *P* > 0.05 in Pearson correlation coefficients

In the first trial, both sides of thoracic PVM, Gmax, and Quad were significantly correlated with all parameters. In the half trial, these and cervical PVM correlated with SpSA and SpPSA (Table 3). In the last trial, cervical PVM, thoracic PVM, Gmax and Quad correlated with SpSVA and SpSA to a similar degree. The correlation between SpPSA and Quad was moderate in the first (right, left; r = 0.53, *P* = 0.0056; r = 0.47, *P* = 0.015) and half trial (r = 0.52, *P* = 0.007; r = 0.49, *P* = 0.012) but weaker in the last trial (r = 0.32, *P* = 0.114; r = 0.35, *P* = 0.0795). Figures 5, 6, and 7 present a summary of correlation coefficients for SpSVA, SpSA, and SpPSA, respectively.

**Table 3:** Correlation analysis of dynamic spinal parameters and muscular activity by iEMG

	SpSVA		SpSA		SpPSA	
First Trial	Rt	Lt	Rt	Lt	Rt	Lt
Cervical PVM	0.32	**0.41**	0.38	**0.50**	**0.47**	**0.54**
Trapezius	-0.06	-0.18	0.00	-0.21	0.14	-0.08
Thoracic PVM	**0.39**	**0.49**	**0.46**	**0.59**	**0.53**	**0.65**
Lumbar PVM	0.21	0.23	0.15	0.18	0.19	0.21
Gmax	**0.53**	**0.66**	**0.45**	**0.64**	0.34	**0.61**
Quadriceps	**0.49**	**0.40**	**0.53**	**0.45**	**0.53**	**0.47**
Half Trial						
Cervical PVM	0.34	**0.44**	**0.40**	**0.52**	**0.47**	**0.54**
Trapezius	-0.13	-0.19	-0.10	-0.23	0.10	-0.09
Thoracic PVM	**0.45**	**0.56**	**0.51**	**0.64**	**0.58**	**0.66**
Lumbar PVM	0.23	0.22	0.17	0.18	0.23	0.20
Gmax	**0.54**	**0.65**	**0.50**	**0.65**	**0.39**	**0.64**
Quadriceps	**0.52**	**0.45**	**0.55**	**0.49**	**0.52**	**0.49**
Last Trial						
Cervical PVM	**0.40**	0.38	**0.42**	**0.43**	**0.44**	**0.45**
Trapezius	-0.10	-0.27	-0.08	-0.30	0.00	-0.20
Thoracic PVM	**0.43**	**0.52**	**0.46**	**0.58**	**0.51**	**0.61**
Lumbar PVM	0.17	0.21	0.09	0.13	0.10	0.02
Gmax	**0.56**	**0.69**	**0.50**	**0.65**	0.35	**0.54**
Quadriceps	**0.40**	**0.43**	**0.44**	**0.47**	0.32	0.35

Muscular activity shown by integrated electromyography (iEMG), which is log transformed for normalized deviation. Significant correlations shown in bold (*P* < 0.05)

*SpSVA* Spinal sagittal vertical axis distance*, SpSA* Spinal sagittal angle*, SpPSA* Spinal-pelvic angle, *Cervical PVM* cervical paravertebral muscle, *Thoracic PVM* thoracic paravertebral muscle, *Lumbar PVM* lumbar paravertebral muscle, *Gmax* gluteus maximus, *F* first trial, *H* half trial, *L* last trial

## Discussion

Our results suggest that spinal balance in patients with ASD gradually deteriorates during the whole course of walking. To compensate for spinal imbalance and keep a horizontal gaze and walking posture, cervical PVM and Gmax are activated gradually during gait. However, thoracic and lumbar PVM did not show correlated activity with spinal balance. We performed correlation analyses between the dynamic spinal parameters deteriorated by walking and muscular activity measured by iEMG. All spinal balance parameters were moderately correlated with cervical PVM, thoracic PVM, Gmax, and Quad in the first and half trials. However, at the end of the last trial, we observed a lowered correlation between Quad activity and SpPSA.

In previous studies, gait analysis has been performed on relatively short distance walking [10, 11, 17–19] or comparing the walking posture between the initiation and cessation of gait [9, 12, 14, 15]. These studies reported that the spinal balance during gait was leaning forward, even in the healthy group [18]; however, the difference from the upright position was significantly larger in the ASD group [19]. While it is known that spinal posture deteriorates when walking in patients with ASD, it remains unknown at which stage during gait this occurs. This study notably found that walking posture differed between the first, half, and last trials, especially whole spinal alignment measured by SpSVA and SpSA, and did not change at any one point during gait. If the posture change occurred in the first stage of walking, 10-m walking could be enough to reveal the functional deformity, which is a deteriorating gait posture over time. The results presented in this study could indicate that the posture after maximum walking was worse than that after shorter walking (as is the case for 10 m), where the static compensation persisted [19]. Thus, this study suggested that short-term walking in the examination room could not reveal functional deformity; hence, it is important to attempt to figure out the actual disability in patients who seemed to have mild or no deformity in the examination room. [20–23]

Walking posture and time-synchronized muscle activity also showed a moderate correlation in this study, especially in the first and half trials. Cervical PVM, thoracic PVM, and Gmax activity increasedwhen spinal sagittal tilt increased during gait. The compensatory mechanism of cervical lordosis in patients with ASD was reported in previous studies using radiographic morphological analysis [20, 22, 23], and the cervical PVM activity observed in this study created cervical hyperlordosis in patients with ASD dynamically as well. In contrast to our hypothesis, no response to increased spinal alignment was evident in the muscle activity of the lumbar PVM. Lower lumbar muscle degeneration is more severe in ASD [12, 24]. The activity of the lumbar PVM might have reflected its fatty infiltration due to severe degeneration, causing the per unit muscle volume measured by surface EMG to be less than the thoracic PVM and Quad.

A previous biomechanical study has reported that spinal sagittal malalignment increases the required muscle activity of the lower limbs [25]. The deteriorated spinal malalignment requires more muscle activity to maintain gait in the last trial compared to the first or half trials, which is consistent with the results of exacerbated spinal malalignment in the first and half trials that cause coordinated Quad muscular activity (Figs. 5–7). However, Quad activity did not significantly increase and was not correlated with deterioration of spinal sagittal malalignment, suggesting that muscle fatigue from long walking duration and inability to increase muscle activity would cause patients with ASD to cease walking. ASD can cause heterogeneous functional limitations other than lower back pain, such as both decreasing muscular output and poor endurance [26]. This dynamic change of Quad activity may explain lower extremity disabilities in patients with ASD.

Rehabilitation is recommended early for patients with ASD, but few studies support this scientific theory. Research data are insufficient, especially because of the long-distance walking difficulty. The current study’s results may indicate the potential of Quad activity rehabilitation to improve maximum walking distance in patients with ASD. Further intervention studies with targeted groups are needed to obtain more robust evidence. In addition, if such a decrease in lower extremity muscle endurance is characteristic of patients with ASD, the cause of this decrease in Quad activity should be explored. Although many surgical treatments have been investigated in ASD, it is difficult to identify the cause or pathophysiology because of the multifactorial nature of the disease. However, examining whether surgical correction alters lower extremity muscle endurance and muscle output can determine the interrelationship between corrective surgery and improvement in muscle output and endurance. This may help predict postoperative outcomes and determine the surgical technique.

There are several limitations of this study. First, the lack of age-adjusted controls was a shortcoming of the analyses. People with spinal malalignment without lower back pain or disability should be included. However, these participants do not show walking disability; therefore, it is difficult to determine the ‘half’ and ‘last’ trials during gait analysis. Second, a small number of patients from a single institution were recruited because of limited accessibility to the gait analysis system. This may have caused selection bias and limitations for the generalization of the results. Third, this study did not analyze coronal imbalance in the included patients. Severe coronal imbalance in patients with ASD causes daily disability and can be related to muscle activity. However, the coronal imbalance is not significant in gait analysis in a previous study [15]. Fourth, we did not analyze the continuous spinal parameters in this study. We need to interpret the entire continuous parameters to determine the percentages of the entire walking distance when posture breakage occurs. Fifth, the reflective markers attached to the clothes may potentially cause measurement errors because of the soft tissue and clothes. Last, we needed to exclude osteoarthritis or other diseases in the lower extremities which could affect walking posture. We confirmed that all patients’ chief complaint was lower back pain and pain in the lower extremities could be dismissed.

In conclusion, the spinal alignment of patients with ASD gradually deteriorated during gait at all trial points of their maximum walking duration. The cervical PVM and Gmax activated, responding to this deterioration in spinal alignment, but the activity of thoracic PVM did not increase. The activity of all measured muscles except the lumbar PVM showed a moderate correlation with dynamic spinal alignment.

## Data Availability

The datasets during and/or analyzed during the current study are available from the corresponding author on reasonable request.

## Abbreviations

3D: Three dimensional
iEMG: Integrated electromyography
PVM: Paravertebral muscle
BMI: Body mass index
C7CSVL: C7 plumb line to central sacral vertical line distance
Cobb: Main Cobb angle
C7SVA: C7 to sacral sagittal vertical axis
TK: Thoracic kyphosis
LL: Lumbar lordosis
PT: Pelvic tilt
PI: Pelvic incidence
TPA: T1 pelvic angle
PI-LL: PI minus LL
Cervical PVM: Cervical paravertebral muscle
Thoracic PVM: Thoracic paravertebral muscle
Lumbar PVM: Lumbar paravertebral muscle
Gmax: Gluteus maximus
Quad: Quadriceps
SpSVA: Spinal sagittal vertical axis distance
SpSA: Spinal sagittal angle
SpPSA: Spinal-pelvic angle
